# Modulation of Insulin Resistance, Dyslipidemia and Serum Metabolome in iNOS Knockout Mice following Treatment with Nitrite, Metformin, Pioglitazone, and a Combination of Ampicillin and Neomycin

**DOI:** 10.3390/ijms23010195

**Published:** 2021-12-24

**Authors:** Hobby Aggarwal, Priya Pathak, Yashwant Kumar, Kumaravelu Jagavelu, Madhu Dikshit

**Affiliations:** 1 Pharmacology Division, CSIR-Central Drug Research Institute, Lucknow 226031, India; hobby.agg@gmail.com (H.A.); priyapathak87@gmail.com (P.P.); kumaraveluj@cdri.res.in (K.J.); 2 Non-Communicable Diseases Division, Translational Health Science and Technology Institute, Faridabad 121001, India; y.kumar@thsti.res.in

**Keywords:** iNOS^-/-^, insulin resistance, dyslipidemia, metabolomic analysis

## Abstract

Oxidative and nitrosative stress plays a pivotal role in the incidence of metabolic disorders. Studies from this lab and others in iNOS^-/-^ mice have demonstrated occurrence of insulin resistance (IR), hyperglycemia and dyslipidemia highlighting the importance of optimal redox balance. The present study evaluates role of nitrite, L-arginine, antidiabetics (metformin, pioglitazone) and antibiotics (ampicillin-neomycin combination, metronidazole) on metabolic perturbations observed in iNOS^-/-^ mice. The animals were monitored for glucose tolerance (IPGTT), IR (insulin, HOMA-IR, QUICKI), circulating lipids and serum metabolomics (LC-MS). Hyperglycemia, hyperinsulinemia and IR were rescued by nitrite, antidiabetics, and antibiotics treatments in iNOS^-/-^ mice. Glucose intolerance was improved with nitrite, metformin and pioglitazone treatment, while ampicillin-neomycin combination normalised the glucose utilization in iNOS^-/-^ mice. Increased serum phosphatidylethanolamine lipids in iNOS^-/-^ mice were reversed by metformin, pioglitazone and ampicillin-neomycin; dyslipidemia was however marginally improved by nitrite treatment. The metabolic improvements were associated with changes in selected serum metabolites-purines, ceramide, 10-hydroxydecanoate, glucosaminate, diosmetin, sebacic acid, 3-nitrotyrosine and cysteamine. Bacterial metabolites-hippurate, indole-3-ethanol; IR marker-aminoadipate and oxidative stress marker-ophthalmate were reduced by pioglitazone and ampicillin-neomycin, but not by nitrite and metformin treatment. Results obtained in the present study suggest a crucial role of gut microbiota in the metabolic perturbations observed in iNOS^-/-^ mice.

## 1. Introduction

Type 2 diabetes is a cardio-metabolic disorder commonly associated with insulin resistance (IR) and dyslipidemia [1]. Oxidative/nitrosative stress due to enhanced reactive oxygen and nitrogen species (RONS) is involved in the pathophysiology of IR, diabetes and obesity. RONS is also crucial for optimal metabolic balance and immune defense [2]. Nitric oxide synthase (NOS) isoforms are the major RONS producing system in the body and their product nitric oxide (NO) plays a pivotal role in maintaining the metabolic homeostasis [3]. Decreased NO bioavailability is linked to the pathogenesis of endothelial dysfunction, hyperlipidemia, obesity and diabetes [4,5,6]. Inducible nitric oxide synthase (iNOS), a calcium independent enzyme, was initially recognised as an inflammatory mediator but has also been identified for its importance in the regulation of metabolism [7,8,9,10]. Studies from this lab [11,12,13] and others [14,15] have demonstrated metabolic perturbations in the obese, insulin resistant and dyslipidemic iNOS^-/-^ mice, which improved partially after nitrite supplementation [13]. iNOS^-/-^ mice also displayed gut microbiota dysbiosis and altered metabolic profile which were reversed by the vancomycin induced depletion of gram-positive bacteria [16]. It suggests the crucial role of optimal redox balance in maintaining the host metabolic homeostasis, and gut microbes composition.

Nitrite, abundantly present in green leafy vegetables, is a precursor of NO and is also known to be protective against cardiovascular disorders and diabetes [17,18]. L-Arginine, a substrate for NOS enzymes for NO generation, nurtures cardiovascular health and endothelium functionality [19]. Metformin, an indirect AMPK activator, improves the glucose metabolism and cellular energy balance in diabetic subjects [20,21]. Pioglitazone, a PPARγ agonist and insulin sensitizer, is routinely used to reduce IR [22]. Germ free (GF) mice are less prone to weight gain, glucose intolerance, insulin resistance, dyslipidemia, obesity and also display altered metabolism in comparison to conventionally used mice [23,24]. Antibiotics-induce gut microbiota depletion protects against diet or genetically-induced obesity and dysmetabolism [25,26,27,28]. Modulation of gut microbiota with norfloxacin and ampicillin in mice improved glucose tolerance [29]. Antibiotic treated HFD fed mice displayed reduced inflammation, oxidative stress, and improved metabolic homeostasis [30]. The studies using germ-free mice [23,24] and antibiotics treated mice [25,26,27,28,29,30] points towards the crucial role of gut microbiota in metabolic homeostasis [31].

Alterations in the redox status and depletion of gut microbiota plays an important role in the metabolic homeostasis [32]. We therefore used a broad-spectrum antibiotic combination (ampicillin-neomycin), and metronidazole (an anaerobic bactericidal agent) to assess their effect on the metabolic homeostasis in iNOS^-/-^ mice. iNOS^-/-^ mice display decrease in total nitrite levels, and supplementing them with nitrite, compensated the NO availability [13]. We therefore also investigated the effect of NO precursors (L-arginine and nitrite), and antidiabetics (metformin and pioglitazone) on glucose intolerance, dyslipidemia and the perturbations in the serum metabolome in iNOS^-/-^ mice.

## 2. Results

### 2.1. Effect of NO Precursors on Systemic Glucose Homeostasis and Dyslipidemia in iNOS^-/-^ Mice

**Figure 1:**
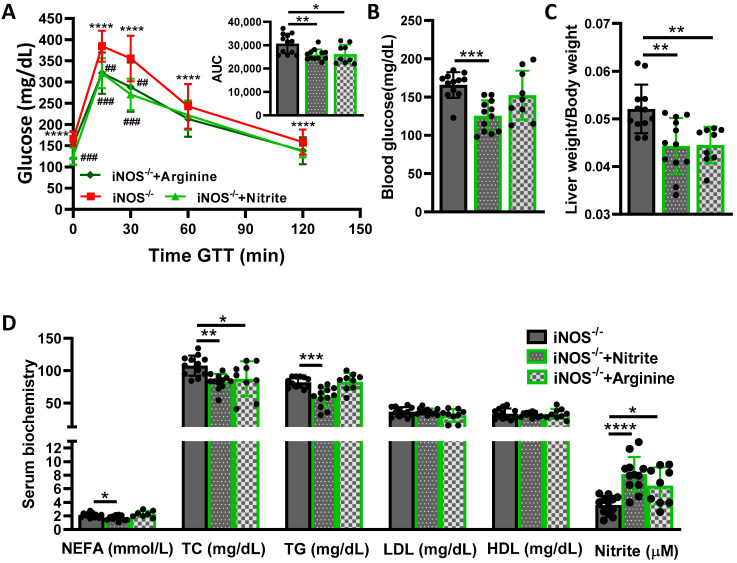
Effect of NO precursors on systemic glucose homeostasis and dyslipidemia in iNOS^-/-^ mice. Systemic glucose and lipid homeostasis in iNOS^-/-^ mice following treatment with nitrite or L-arginine. (**A**) Intraperitoneal glucose tolerance test (GTT) with AUC calculated from IPGTT data, (**B**) Fasting blood glucose levels, (**C**) Relative liver-body weight ratio and (**D**) Serum lipids and total nitrite levels. Data are represented as mean ± SD (*n* = 7–12). * *p* < 0.05, ** *p* < 0.01, *** *p* < 0.001 and **** *p* < 0.0001 between indicated groups. ## *p* < 0.01 and ### *p* < 0.001 and vs. iNOS^-/-^ in GTT curve.

Chow fed iNOS^-/-^ mice were glucose intolerant (Figure S1A) and hyperglycemic (Figure S1B) as compared to the age matched WT controls, along with reduced circulating level of total nitrite content (Figure S1D). Treatment with nitrite or L-arginine to enhance the NO availability in iNOS^-/-^ mice, partially improved glucose intolerance as assessed by GTT (Figure 1A). Hyperglycemia was reversed by nitrite but not by L-arginine treatment in iNOS^-/-^ mice (Figure 1B). The increase in liver-body weight ratio in iNOS^-/-^ mice (Figure S1C) was reverted by the treatment with nitrite and L-arginine (Figure 1C). Circulating NEFA, total cholesterol, triglycerides and LDL content were significantly enhanced in iNOS^-/-^ mice, while HDL levels were comparable to WT (Figure S1D). Nitrite treatment reduced the circulating NEFA, TC and TG levels in iNOS^-/-^ mice, while LDL and HDL levels were not altered. On the other hand, treatment with L-arginine marginally decreased the TC levels without any effect on other lipids (NEFA, TG, LDL and HDL; Figure 1D). Total nitrite levels in the serum were completely restored by nitrite treatment, while treatment with L-arginine marginally enhanced the nitrite content (Figure 1D). These results suggest that nitrite treatment partially improved the glucose intolerance and dyslipidemia in iNOS^-/-^ mice with reversal in NO availability. L-Arginine supplementation marginally improved the NO levels and glucose intolerance, but had no effect on hyperglycemia and dyslipidemia. This points towards the involvement of additional factors other than NO in glucose intolerance and dyslipidemia observed in iNOS^-/-^ mice. The effects of nitrite treatment on metabolic parameters were also checked in WT mice. Glucose tolerance, serum glucose, lipids and nitrite levels remained unaltered in WT mice following treatment with nitrite (Figure S2A–D, Table S1).

### 2.2. Effect of Anti-Diabetics on Systemic Glucose Homeostasis and Dyslipidemia in iNOS^-/-^ Mice

**Figure 2:**
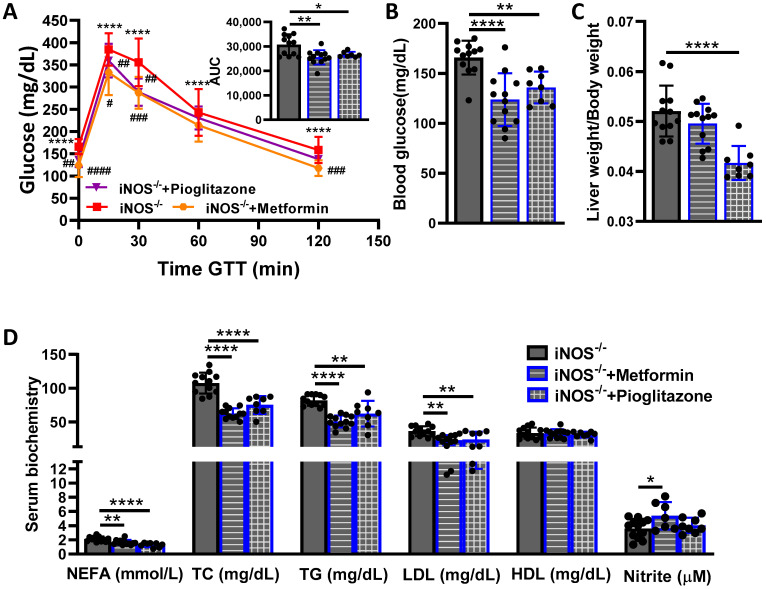
Effect of anti-diabetics on systemic glucose homeostasis and dyslipidemia in iNOS^-/-^ mice. Systemic glucose and lipid homeostasis in iNOS^-/-^ mice after treatment with metformin or pioglitazone. (**A**) Intraperitoneal glucose tolerance test (GTT) with AUC calculated from IPGTT data, (**B**) Fasting blood glucose levels, (**C**) Relative liver-body weight ratio and (**D**) Serum lipids and total nitrite levels. Data are represented as mean ± SD (*n* = 7–12). * *p* < 0.05, ** *p* < 0.01 and **** *p* < 0.0001 between indicated groups. # *p* < 0.05, ## *p* < 0.01, ### *p* < 0.001 and #### *p* < 0.0001 vs. iNOS^-/-^ in GTT curve.

Treatment of iNOS^-/-^ mice with metformin or pioglitazone partially improved the glucose intolerance as assessed by GTT (Figure 2A) and reversed the hyperglycemia (Figure 2B). The change in liver-bodyweight ratio was reversed by pioglitazone treatment in iNOS^-/-^ mice (Figure 2C). Circulating NEFA, TC, TG and LDL levels were rescued by metformin and pioglitazone treatment in iNOS^-/-^ mice, with no effect on HDL levels (Figure 2D). Total nitrite levels in the serum were marginally enhanced by the metformin intervention with no effect of pioglitazone treatment in iNOS^-/-^ mice (Figure 2D). These results suggest that anti-diabetics partially improved the glucose intolerance and reversed the dyslipidemia in iNOS^-/-^ mice. Glucose tolerance, serum glucose, lipids and nitrite levels remained unaltered in WT mice treated with metformin, with only marginal decrease in TC levels (Figure S2A–D, Table S1).

### 2.3. Effect of Antibiotics on Systemic Glucose Homeostasis and Dyslipidemia in iNOS^-/-^ Mice

**Figure 3:**
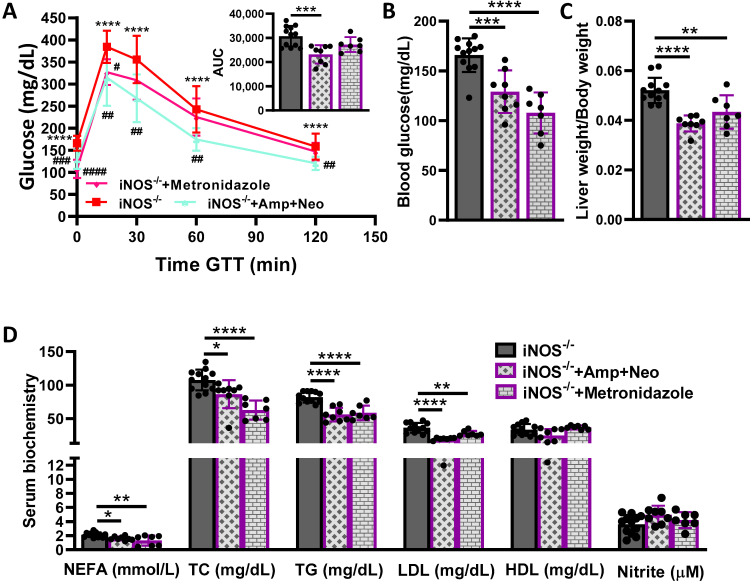
Effect of antibiotics on systemic glucose homeostasis and dyslipidemia in iNOS^-/-^ mice. Systemic glucose and lipid homeostasis in iNOS^-/-^ mice upon treatment with ampicillin-neomycin combination and metronidazole. (**A**) Intraperitoneal glucose tolerance test (GTT) with AUC calculated from IPGTT data, (**B**) Fasting blood glucose levels, (**C**) Relative liver-body weight ratio and (**D**) Serum lipids and total nitrite levels. Data are represented as mean ± SD (*n* = 7–12). * *p* < 0.05, ** *p* < 0.01, *** *p* < 0.001 and **** *p* < 0.0001 between indicated groups. # *p* < 0.05, ## *p* < 0.01, ### *p* < 0.001 and #### *p* < 0.0001 vs. iNOS^-/-^ in GTT curve.

Broad spectrum antibiotic combination of ampicillin and neomycin, was used to deplete the gut microbiota in iNOS^-/-^ mice, which completely reversed the glucose intolerance (Figure 3A). Metronidazole, was used to deplete the anaerobic bacteria in iNOS^-/-^ mice, which did not affect glucose intolerance (Figure 3A). Treatment with both ampicillin-neomycin combination and metronidazole improved the hyperglycemia in iNOS^-/-^ mice (Figure 3B). The change in liver-body weight ratio was improved by ampicillin-neomycin combination as well as metronidazole treatment in iNOS^-/-^ mice (Figure 3C). Circulating NEFA, TC, TG and LDL levels in iNOS^-/-^ mice were rescued fully by ampicillin-neomycin combination and metronidazole treatment (Figure 3D). Total nitrite levels in the serum remained unaltered after ampicillin-neomycin combination or metronidazole treatment in iNOS^-/-^ mice (Figure 3D). These results point towards the substantial effect of broad-spectrum antibiotic-ampicillin-neomycin combination on glucose intolerance and circulating lipids in iNOS^-/-^ mice as compared to other interventions. Glucose tolerance, lipids and nitrite levels remained unaltered in WT mice treated with ampicillin-neomycin combination with declined blood glucose levels (Figure S2A–D, Table S1). These observations suggest the pronounced effect of antibiotic-ampicillin-neomycin combination on glucose homeostasis.

### 2.4. Modulation of Insulin Homeostasis in iNOS^-/-^ Mice by Nitrite, Metformin, Pioglitazone and a Combination of Ampicillin and Neomycin

**Figure 4:**
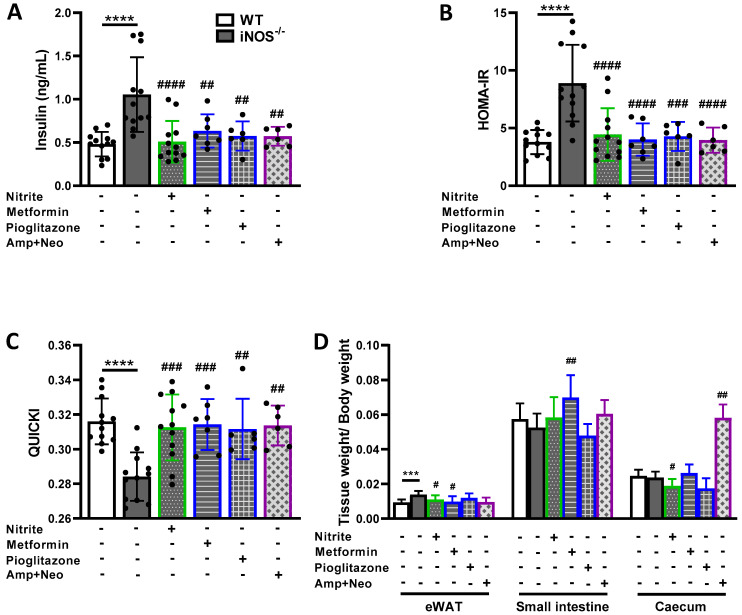
Modulation of insulin homeostasis in iNOS^-/-^ mice by nitrite, metformin, pioglitazone, and a combination of ampicillin-neomycin. (**A**) Fasting serum insulin levels; Indices of insulin resistance (**B**) HOMA-IR and insulin sensitivity (**C**) QUCIKI (*n* = 6–12). (**D**) Relative tissue weights (*n* = 4–12). Data are represented as mean ± SD. *** *p* < 0.001 and **** *p* < 0.0001 vs. WT; # *p* < 0.05, ## *p* < 0.01, ### *p* < 0.001 and #### *p* < 0.0001 vs. iNOS^-/-^.

Further experiments were done on iNOS^-/-^ mice treated with NO precursor-nitrite, standard antidiabetic drugs-metformin, pioglitazone, and antibiotic-ampicillin-neomycin combination. Treatment of iNOS^-/-^ mice with L-arginine had no effect on systemic lipid and glucose levels, while metronidazole treatment had no effect on glucose intolerance. We therefore did not use these interventions (L-arginine and metronidazole) in subsequent studies. Hyperinsulinemia, IR (as assessed by HOMA-IR) and insulin sensitivity (as assessed by QUICKI) were restored completely by treatment with nitrite, metformin, pioglitazone and ampicillin-neomycin combination in iNOS^-/-^ mice (Figure 4A–C). HOMA-B remained unaltered by metformin, pioglitazone and a combination of ampicillin-neomycin treatment in iNOS^-/-^ mice; while, it was decreased by nitrite treatment (Figure S3D). Insulin levels and HOMA-B were unaltered by nitrite, metformin, ampicillin-neomycin combination treatment in WT mice; while HOMA-IR was reduced and QUICKI was elevated by ampicillin-neomycin combination which points towards the marked role of antibiotics in glucose and insulin homeostasis (Figure S2E,F; Table S1). Overall, IR was improved by nitrite, metformin, pioglitazone and ampicillin-neomycin combination in iNOS^-/-^ mice.

The enhanced body weight of iNOS^-/-^ mice did not display any significant change after nitrite, metformin or pioglitazone treatment. However, decrease in body weight was observed at the first and third week of ampicillin-neomycin treatment with no significant overall change during the study period (Figure S3A). Food intake was also not altered upon treatment with nitrite, metformin, pioglitazone, and ampicillin-neomycin combination in iNOS^-/-^ mice (Figure S3B). Further, total bacteria levels checked by qPCR analysis using 16S rRNA gene specific primer in faeces confirmed the gut microbiota depletion in ampicillin-neomycin combination treated iNOS^-/-^ mice along with enhanced caecum weight ratio (Figure 4D). While, bacterial 16S levels were not altered by other treatments (Figure S3C). Relatively epididymal white adipose tissue weight (eWAT) was increased in iNOS^-/-^ mice and was reduced by nitrite and metformin treatment. Small intestine weight was enhanced by metformin in iNOS^-/-^ mice (Figure 4D). While spleen, kidney, heart, brown adipose tissue (BAT) and colon weight remained unaltered by nitrite, metformin, pioglitazone and ampicillin-neomycin combination in iNOS^-/-^ mice except decreased heart weight ratio by ampicillin and neomycin combination in both iNOS^-/-^ and WT mice, and increased BAT weight ratio by pioglitazone treatment in iNOS^-/-^ mice (Figure S3D, Table S1). Weight of the small intestine and caecum were enhanced by nitrite and by the combination of ampicillin-neomycin treatment respectively in WT mice (Table S1). These results suggest than insulin homeostasis was altered in iNOS^-/-^ mice upon ampicillin-neomycin combination, metformin, pioglitazone and nitrite treatment, without affecting the body weight and food consumption.

### 2.5. Alterations in the Serum Metabolome of iNOS^-/-^ Mice following Treatment with Various Interventions

**Figure 5:**
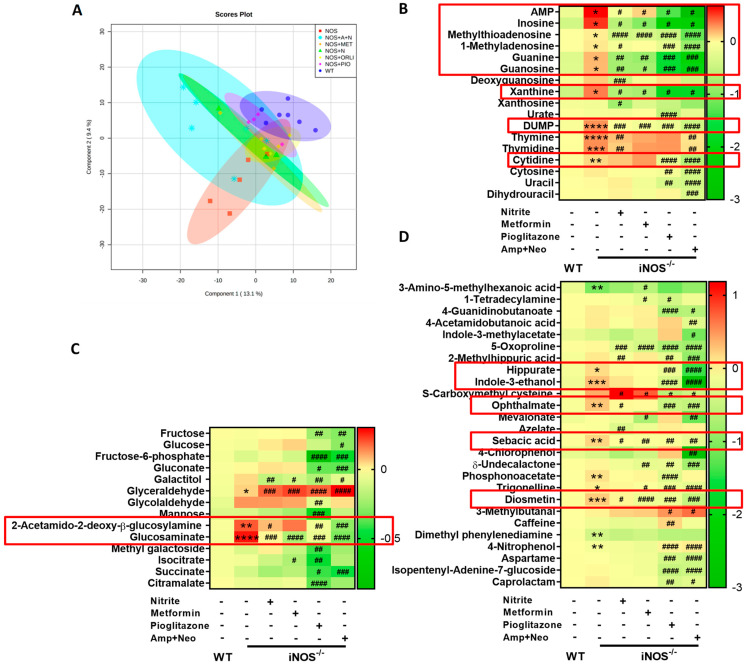
Alterations in the serum metabolome of iNOS^-/-^ mice following treatment with various interventions. Serum metabolomic analysis in chow fed WT, iNOS^-/-^ and iNOS^-/-^ mice treated with various interventions in ESI (+) mode (**A**) PLS-DA score plot. Heat map of differential metabolites identified by metabolomics analysis related to (**B**) Nucleic acids metabolism, (**C**) Carbohydrate metabolism and (**D**) Miscellaneous/microbiota derived metabolites. Data are represented as mean (*n* ≥ 4). * *p* < 0.05, ** *p* < 0.01, *** *p* < 0.001, **** *p* < 0.0001 vs. WT; # *p* < 0.05, ## *p* < 0.01, ### *p* < 0.001 and #### *p* < 0.0001 vs. iNOS^-/-^.

PCA score scatter plot of serum metabolites from WT, untreated iNOS^-/-^ mice and nitrite, metformin, pioglitazone, and ampicillin-neomycin treated iNOS^-/-^, were distinctly separated, which have been represented in ESI positive mode in Figure 5A. Out of 255 annotated metabolites, 40 were significantly increased in iNOS^-/-^ mice and 15 were significantly decreased. Out of 40 metabolites which were upregulated in iNOS^-/-^ mice, 27 were significantly reduced by nitrite, 28 by metformin, 35 by pioglitazone, and 36 by ampicillin-neomycin treatments. iNOS^-/-^ mice displayed enhanced purine (AMP, inosine, methylthioadenosine, 1-methyladenosine, guanine, guanosine, xanthine) and pyrimidine metabolites [deoxyuridine monophosphate (dUMP), thymine, thymidine, cytidine]. Purine metabolites and DUMP were reversed by nitrite, metformin, pioglitazone and ampicillin-neomycin treatments in iNOS^-/-^ mice. Thymine and thymidine (pyrimidine) were decreased by treatment with nitrite and, ampicillin-neomycin combination in iNOS^-/-^ mice. Cytidine was decreased by pioglitazone and a combination of ampicillin-neomycin (Figure 5B). Purine and pyrimidine metabolites were decreased by metformin, and ampicillin-neomycin combination in WT mice (Figure S4A). These results suggest that purine metabolites were reversed by all these interventions in iNOS^-/-^ mice, whereas, pyrimidine metabolites were improved by nitrite and ampicillin-neomycin combination. Enhanced glucosaminate in iNOS^-/-^ mice were reversed by treatment with nitrite, metformin, pioglitazone and, ampicillin-neomycin combination. 2-Acetamido-2-deoxy-β-glucosylamine was increased in iNOS^-/-^ mice and was decreased by treatment with nitrite, pioglitazone and, ampicillin-neomycin combination (Figure 5C). Glycolytic and Kreb’s cycle intermediates were decreased in ampicillin-neomycin treated WT mice (Figure S4B). Hippurate, indole-3-ethanol (bacterial metabolites) and ophthalmate (oxidative stress marker) were augmented in iNOS^-/-^ mice and were normalized by the treatment with pioglitazone, and ampicillin-neomycin combination. Diosmetin (flavone) and sebacic acid in iNOS^-/-^ mice was reduced by the treatment with nitrite, metformin, pioglitazone, and ampicillin-neomycin combination (Figure 5D). Bacterial derived metabolites- hippurate, indole-3-ethanol and ophthalmate were significantly (*p* < 0.001) lowered by ampicillin-neomycin combination only in WT and not by nitrite or metformin (Figure S4C). These results suggest that ampicillin-neomycin combination showed distinct changes in the bacterial-derived metabolites in both WT and iNOS^-/-^ mice.

**Figure 6:**
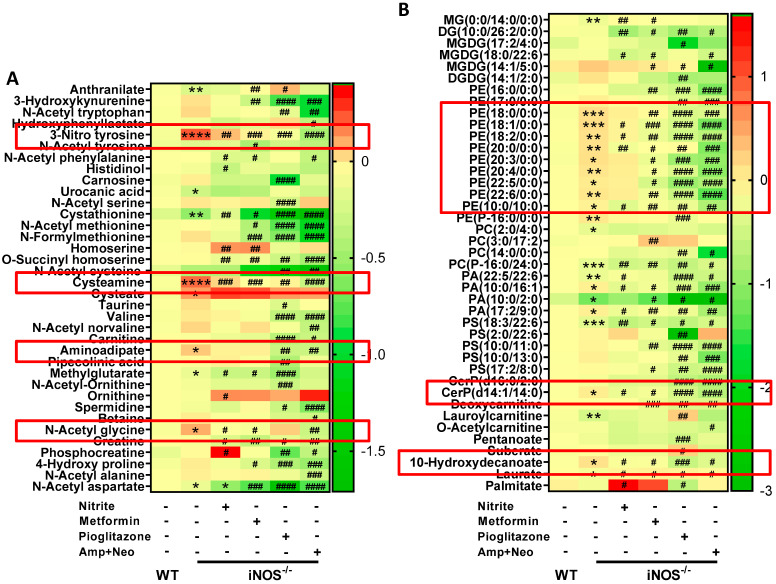
Alterations in the amino acid and lipid metabolites in iNOS^-/-^ mice following treatment with various interventions. Heat map of differential metabolites found by metabolomics analysis related to (**A**) Amino acids metabolism and (**B**) Lipid metabolism. Data are represented as mean (*n* ≥ 4). * *p* < 0.05, ** *p* < 0.01, *** *p* < 0.001, **** *p* < 0.0001 vs. WT; # *p* < 0.05, ## *p* < 0.01, ### *p* < 0.001 and #### *p* < 0.0001 vs. iNOS^-/-^. MG: Monoacylglycerols, DG: Diacylglycerols, MGDG: monogalactosyldiacylglycerol, DGDG: digalactosyldiacylglycerol, PE: Phosphatidylethanolamine, PC: Phosphatidylcholine, PA: Phosphatidic acid, PS: Phosphatidylserine and CerP: Ceramide phosphate.

Amino acid metabolites- 3-nitrotyrosine, cysteamine, cysteate, aminoadipate and N-acetylglycine were increased in iNOS^-/-^ mice. 3-Nitrotyrosine and cysteamine were decreased by nitrite, metformin, pioglitazone, and ampicillin and neomycin combination treatment in iNOS^-/-^ mice. Enhanced cysteate levels in iNOS^-/-^ were not altered by any of the used interventions. While aminoadipate was decreased by pioglitazone, and ampicillin-neomycin combination in iNOS^-/-^ and by ampicillin-neomycin combination in WT (Figures 6A and S5A). The results obtained suggest a correlation between gut bacteria and aminoadipate, as it was downregulated in both WT and iNOS^-/-^ by ampicillin-neomycin combination treatment. PE lipids, PE to PC ratio, ceramide and 10-hydroxydecanoate were increased in iNOS^-/-^ mice, which exhibited decrease in PC lipids, PS lipids, laurate and lauroyl carnitine. Ceramide and 10-hydroxydecanoate were decreased by treatment with nitrite, metformin, pioglitazone, or ampicillin-neomycin combination in iNOS^-/-^ mice. PE lipids were decreased by metformin, pioglitazone, and ampicillin-neomycin combination in iNOS^-/-^ mice; while nitrite treatment had only marginal effect (Figure 6B). PE lipids, ceramides and 10-hydroxydecanoate were decreased by ampicillin-neomycin in WT mice, remained unaltered by nitrite and metformin treatment. PC, PA, PS lipids were reduced by nitrite, metformin, and ampicillin-neomycin combination in WT mice (Figure S5B). The metabolites which were downregulated in iNOS^-/-^ mostly remained unchanged or reduced after various interventions, except laurate, lauroyl carnitine, 4-nitrophenol and anthranilate. Laurate was upregulated by nitrite, metformin, pioglitazone, and ampicillin-neomycin combination; lauroyl carnitine and anthranilate by pioglitazone; and 4-nitrophenol by pioglitazone, and ampicillin-neomycin combination. The metabolites that were augmented in iNOS^-/-^ mice seems to be more crucial for metabolic discrepancies. Overall, nitrite treatment had no effect on PE lipids, while metformin has no effect on pyrimidine metabolites. Microbe-derived or metabolized-hippurate, indole-3-ethanol, and aminoadipate were not altered by both nitrite and metformin. Both host and microbe-derived metabolites, in iNOS^-/-^ mice treated with ampicillin-neomycin combination displayed similar trend as control with complete rescue in glucose intolerance, insulin resistance and dyslipidemia. These results suggest that for complete reversion of metabolic perturbations in iNOS^-/-^ mice metabolites such as hippurate, indole-3-ethanol, aminoadipate, ophthalmate and pyrimidine metabolites are crucial along with purines, PE lipids, ceramide, 10-hydroxydecanoate, glucosaminate, sebacic acid, diosmetin, 3-nitrotyrosine and cysteamine.

### 2.6. Association of Serum Metabolites with Metabolic Profile of iNOS^-/-^ Mice after Treatment with Various Interventions

**Figure 7:**
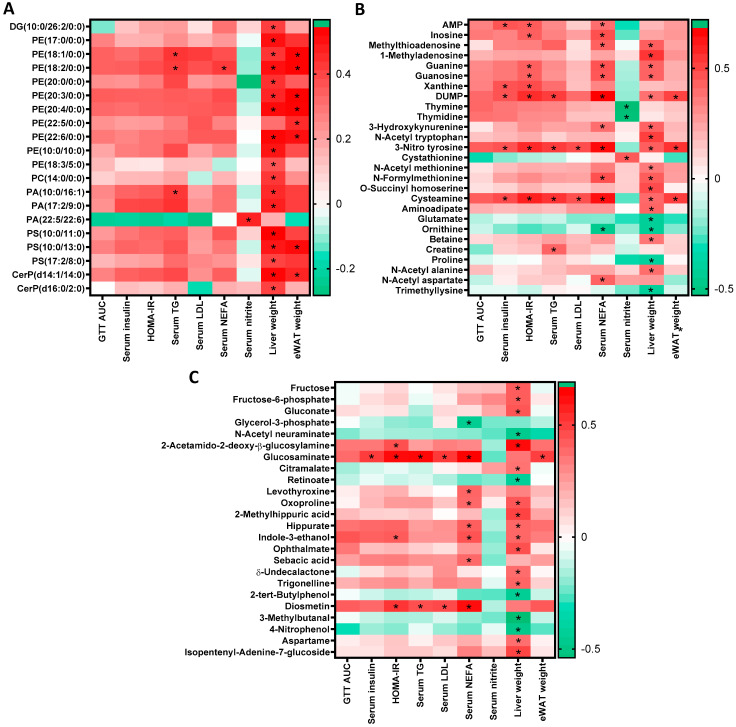
Association of serum metabolites with metabolic profile of iNOS^-/-^ mice following treatment with various interventions. Heat map and analysis based on Pearson’s correlation coefficients suggest direct correlation between metabolic biomarkers and serum metabolites from WT, iNOS^-/-^ mice following treatment with various interventions. (**A**) Lipid metabolism, (**B**) Nucleic acid and amino acid metabolism and (**C**) Carbohydrate metabolism, vitamins and hormones metabolism, and miscellaneous/microbiota derived metabolites. * *p* < 0.05 represent significant correlations between metabolic biomarker and serum metabolites. Green color represents negative and red positive correlations.

The phenotypic, biochemical, functional and metabolic analysis suggests that insulin resistant and dyslipidemic iNOS^-/-^ mice showed marked improvement in metabolism upon treatment with nitrite, metformin, pioglitazone or a combination of ampicillin-neomycin. Pearson’s correlation was therefore used to identify the serum metabolites which strongly correlated with the metabolic biomarkers in these animals. Lipid species-PEs, PAs, PSs and ceramides correlated positively with liver and adipose tissue weight suggesting their association with enhanced lipid accumulation (Figure 7A). Purine metabolites also correlated positively with IR and dyslipidemia (circulating lipids and liver weight). Pyrimidine metabolites-thymine and thymidine showed inverse association with serum nitrite levels, while cystathionine was correlated directly (Figure 7B). Pyrimidine-DUMP, amino acid metabolites-3-nitrotyrosine and cysteamine; carbohydrate metabolites-glucosaminate; and flavone-diosmetin were found to be positively correlated with IR and dyslipidemia (Figure 7B,C). Bacterial metabolites-hippurate, indole-3-ethanol and sebacic acid correlated positively with dyslipidemia (Figure 7C). These results suggest that select serum metabolites exhibited strong association with specific metabolic biomarkers. Metabolites that were altered in iNOS^-/-^ mice, correlated strongly with dyslipidemia, IR and glucose intolerance and most of them including the bacterial-derived metabolites were rescued by the treatment with ampicillin-neomycin combination. The data obtained from ampicillin-neomycin treated iNOS^-/-^ mice suggest collective role of both host and microbe-derived serum metabolites in reverting the glucose tolerance, IR, dyslipidemia.

## 3. Discussion

Reduced NO bioavailability coupled with oxidative and nitrosative stress is linked to IR and obesity [18,33,34]. Mice fed with low nitrite diet also displayed IR, glucose intolerance and dyslipidemia [5]. Non-specific NOS inhibition led to enhanced circulating lipids and hepatic fat deposition in rats [35,36], and metabolic syndrome in mice [37]. Single (eNOS) [17], double (eNOS/nNOS) [38] and triple (eNOS, nNOS and iNOS) [39] NOS KO mice also displayed metabolic disruptions. Intriguingly, iNOS gene genetic polymorphism (14-repeat allele) is associated with enhanced iNOS activity conferring selective advantage to diabetic individuals against nephropathy and retinopathy [40,41]. On the other hand, iNOS knockout had systemic IR and metabolic disturbances [11,12,13,14,15]. Effect of nitrite supplementation has been well studied in eNOS [17,42] and nNOS knockout mice [43] but not in iNOS^-/-^ mice. Recently, our lab has demonstrated improvement in IR and insulin signaling by nitrite supplementation in iNOS^-/-^ mice due to compensation of reduced NO availability [13]. Present study was thus undertaken to assess the effect of various interventions such as NO precursors, antidiabetics and antibiotics, on glucose utilization, dyslipidemia and serum metabolome in iNOS^-/-^ mice. Glucose intolerance, IR and dyslipidemia were improved by the nitrite and anti-diabetic drugs treatment but were completely rescued by gut microbiota depletion using a combination of ampicillin-neomycin. Results obtained suggest role of both host and microbe-derived metabolites in the KO mice thus establishing a link between gut microbiota and metabolic perturbations.

Low nitrite levels in iNOS^-/-^ mice [11,44], as expected, were compensated by nitrite supplementation with improvement in hyperglycemia, glucose utilization and dyslipidemia. These observations corroborated with the beneficial effect of nitrite/nitrate observed on glucose and insulin homeostasis in diabetic KKA^y^ mice [45], eNOS^-/-^ mice [17,42], HFD fed diabetic rats [46], db/db mice [47] and also in WT mice [48]. LDL levels were not lowered by nitrite treatment [5,46] due to enhanced hepatic expression of PCSK9 along with LDLR [13]. In addition, increased dose of nitrite supplementation also did not rescue the LDL levels (data not shown). In absence of iNOS, it was speculated that NO can be compensated by the other NOS enzymes in the presence of L-Arginine. It did improve glucose utilization with no improvement in dyslipidemia due to marginal increase in NO levels in iNOS^-/-^ mice. Improvement in glucose homeostasis was also observed in HFD fed rats [49], hyper-lipidemic hamsters [50] and humans [51]. Improvement in glucose homeostasis after enhancing NO levels in iNOS^-/-^ mice suggests the crucial role of homoeostatic NO/iNOS in metabolic regulation. As dyslipidemia and glucose utilization were not rescued completely despite total reversal of NO levels, it suggests the involvement of additional regulators in the metabolic perturbations observed in iNOS^-/-^ mice other than reduced NO levels.

Routinely used insulin sensitizers metformin and pioglitazone, improved the glucose utilization and dyslipidemia in iNOS^-/-^ mice along with rescued circulating LDL which was not affected by NO sufficiency. Metformin is known to improve cellular energy balance via AMPK by enhancing glucose homeostasis, insulin sensitivity, and by suppressing glucose production [20,21] and circulating lipids [52]. Pioglitazone a PPARγ agonist, decreased IR in major metabolic tissues [22], dyslipidemia [53], and the hepatic lipid accumulation in iNOS^-/-^ mice as suggested by decreased liver/body weight ratio. These findings support the beneficial effect of anti-diabetics on IR and dyslipidemia in iNOS^-/-^ mice with partial improvement in glucose homeostasis. Herrera et al. showed equipotent metabolic effects of nitrate and metformin on glucose-insulin homeostasis and cardiac hypertrophy in the mice treated with NOS inhibitor and fed on HFD via improved AMPK signaling and reduced oxidative stress [37]. Similarly, in the present study we also observed decreased liver-body weight ratio in nitrite but not in metformin treated iNOS^-/-^ mice.

Recent studies on germ-free [23,24] and antibiotics-treated rodents [25,27,29,30,54,55,56,57] suggest the crucial role of gut microbiota on host metabolism [58]. The absence of gut microbiota in germ free mice [23,24] or depletion of microbiota by antibiotics treatment in WT [55,56,57] and diet induced obese mice [25,27,29,30,54] not only reduced glucose and insulin levels but also improved glucose intolerance and insulin insensitivity. Similarly, we found complete recovery of glucose intolerance, IR and dyslipidemia in the iNOS^-/-^ mice treated with broad spectrum antibiotics, while other interventions used in the present study were not so efficacious. Both gram-positive and negative bacteria are known to be involved in the metabolic disorders [59,60]. Decreased bacterial levels and caecum engorgement are the signature markers of antibiotics treatment which were also observed by us and others [57]. The present study also supports a crucial role of the gut microbiota in metabolic perturbation. The heart weight to body weight ratio was decreased after treatment with ampicillin-neomycin combination in WT and iNOS^-/-^ mice, without any change in the absolute heart weight. There are few reports suggesting a link between gut microbiota and heart failure [61,62]. Moreover, calorie restriction also results in the reduction of body weight and leptin levels [63]. Further and detailed studies are however required to establish this link in iNOS mice.

Nucleic acid and lipid metabolism are the principal disordered pathways in iNOS^-/-^ mice. Purine metabolites were enhanced in iNOS^-/-^ mice with similar reports in the patients of diabetes, indicating disease progression, enhanced hepatic glucose production and oxidative damage [64,65]. Increase in the levels of purine metabolites was observed in the erythrocytes of diabetic patients indicating higher turnover of nucleotides and hyper metabolism [65]. Pyrimidine metabolism correlates well with type 2 diabetes [66] and was found to be enhanced in iNOS^-/-^ mice. This enhancement was reversed by nitrite and ampicillin-neomycin intervention thus suggesting an association between NO and gut microbiota. Enhanced PE to PC ratio has been associated with NAFLD/NASH in humans with higher propensity of liver damage and altered glycemic perturbations [67,68]. Moreover, hydroxydecanoate, associated with obesity and diabetes, [69] and PE/PC ratio were improved after treatment with anti-diabetics, and antibiotics in iNOS^-/-^ mice but not by nitrite treatment suggesting its limited effect on dyslipidemia.

Increase in the oxidative stress marker, 3-nitrotyrosine along with ophthalmate, in iNOS^-/-^ mice was similar to dyslipidemic and diabetic rodent models [70,71,72] which could be independent of iNOS [73,74]. Ampicillin-neomycin completely rescued the change in oxidative stress markers and glucose homeostasis which is similar to the previous reports [29,30]. Glucosaminate associated with pentose phosphate pathway and microbial metabolism in diverse environments was upregulated in iNOS^-/-^ mice and it was rescued by nitrite or anti-diabetic drugs treatments. 2-Aminoadipate has been associated with diabetes, obesity and metabolic syndrome risk in humans [75,76] and this diabetogen was also augmented in iNOS^-/-^ mice but reverted by only pioglitazone and ampicillin-neomycin treatment. The metabolites that were decreased in iNOS^-/-^ mice were not altered by treatments suggesting their direct association with iNOS gene knockdown in the host.

Cysteamine [77] and diosmetin [78] abrogate oxidative stress and improve insulin and redox signaling, were enhanced in iNOS^-/-^ mice and were reduced by nitrite, anti-diabetics and antibiotics. Sebacic acid, found augmented in the diabetics [79] and NAFLD patients [80], was decreased following treatment with various interventions in iNOS^-/-^ mice. Hippurate, linked with microbial gene richness [81], is contributed by gram-positive bacteria [82] is also an early biomarker of IR and diabetes [83]. Indole-3-ethanol, a bacterial derived catabolite of tryptophan [84] was increased in iNOS^-/-^ mice. Change in these bacterial metabolite was reversed by pioglitazone and ampicillin-neomycin treatment suggesting the possible association of gut microbiota with IR in iNOS^-/-^ mice. The absolute reversal of glucose intolerance, IR, dyslipidemia and perturbations in the host as well as in microbial derived metabolites in iNOS^-/-^ mice with ampicillin-neomycin intervention establish that gut bacteria are crucial drivers of the metabolic phenotype in these mice. Increase in the intestinal length and weight was observed in the metformin treated iNOS^-/-^ mice. Metformin is known to increase the relative abundance of gram-negative bacteria, *Akkermansia muciniphila* [85] which was also increased in iNOS^-/-^ mice. On the contrary, effect of pioglitazone on human gut microbiota is not reported but it is shown to decrease the microbial diversity and shifts the beta diversity in diabetic animal models [86,87]. Our results point towards the possible role of gut microbiota in pioglitazone-mediated improvement in metabolic alterations in iNOS^-/-^ mice. The iNOS KO mice might be useful to further understand the distinct insulin resistant state with obese phenotype, as gram-positive infections are more prevalent in the western countries [88]. Animal to human translational success however also needs to be established.

The present study thus demonstrates collective role of host and microbe-derived metabolites in glucose intolerance, IR, dyslipidemia, which was reversed by the gut microbiota depletion by the combination of ampicillin-neomycin treatment. The study establishes an association of gut microbiota in the metabolic perturbations in the iNOS KO mice.

## 4. Materials and Methods

### 4.1. Mice and Diet

Age matched, twelve to thirteen weeks old, male C57BL/6J (WT) mice and iNOS knockout (iNOS^-/-^; Jackson Laboratory, Bar Harbor, ME, USA; 002609) on C57BL/6J background were bred and maintained at 24 ± 2 °C in IVC cages (Tecniplast, Buguggiate, VA, Italy). All procedures were approved by Institutional Animal Ethics Committee of CSIR-CDRI (IAEC/2014/43/Renew dated 4 November 2016) in accordance with CPCSEA guidelines. Both WT and iNOS^-/-^ mice were maintained on chow diet (1320, Altromin, Lage, North Rhine-Westphalia, Germany) with water *ad libitum*. iNOS^-/-^ mice were supplemented with NO precursors-sodium nitrite (NaNO_2_, 50 mg/L) [13] and L-arginine (1% *w*/*v*) [89] in drinking water for 5 weeks. Anti-diabetics-metformin (350 mg/kg) [90] and pioglitazone (10 mg/kg) [90] were administered orally to iNOS^-/-^ mice for 5 weeks. Antibiotics-ampicillin (1 g/L) and neomycin (0.5 g/L) combination [91], and metronidazole (1 g/L) [92] were administered via drinking water for 4 weeks for depletion of majority of gut microbiota and anaerobic bacteria respectively. Body weight and food consumption was measured weekly from day zero to the completion of study.

### 4.2. Glucose Tolerance Test (GTT)

Glucose tolerance test was performed by administration of 2 g/kg D-Glucose by intraperitoneal (i.p.) route to 6 h fasted mice. Blood glucose was monitored at 0, 15, 30, 60 and 120 min after administration of glucose using Accu-Chek glucometer (Roche Diagnostics, Mumbai, Maharashtra, India) and area under the curve (AUC) was calculated [11].

### 4.3. Serum Biochemistry

Mice were fasted for 6 h, blood was collected from retro-orbital plexus and serum separated. Total cholesterol (TC), triglycerides (TG), low density lipoproteins (LDL), high density lipoproteins (HDL) and non-esterified fatty acids (NEFA) lipids estimation was performed in the serum using kits from Randox, Crumlin, Co. Antrim, UK [12]. Serum insulin levels were measured using kit from Crystal Chem, Elk Grove Village, IL, USA as per manufacturer’s instructions. Fasting blood glucose levels and serum insulin levels were used to calculate the indices of IR-HOMA-IR, β-cell functionality-HOMA-B and insulin sensitivity-QUICKI as per the formulae used by other investigators [93].

### 4.4. Total Nitrite Estimation

Total nitrite levels (nitrate and nitrite) were estimated in serum (100 μL) using pre-activated cadmium pellets and vigorous shaking for 4 h at room temperature for reducing nitrate to nitrite. Equal volumes (1:1) of supernatant and Griess reagent were incubated for 30 min (37 °C in dark) and absorbance was taken at 545 nm. Total nitrite concentration of samples was calculated using sodium nitrite as standard [11].

### 4.5. Relative Bacterial Levels Estimation in Faeces Using qPCR

For quantification of relative faecal bacterial load, total DNA was isolated from 125 mg of faeces using QIAamp DNA Stool Mini Kit (Qiagen, Hilden, Germany) as per the manufacturer’s instructions [94]. DNA was then subjected to quantitative PCR using DyNAmo ColorFlash SYBR Green qPCR kit with universal 16S rRNA forward 5′-TCCTACGGGAGGCAGCAGT and reverse 5′-GGACTACCAGGGTATCTAATCCTGTT primers and relative bacterial 16S levels were compared.

### 4.6. Metabolomics Analysis

#### 4.6.1. Sample Preparation

Lyophilised serum (100 µL) samples were reconstituted in 200 µL methanol, 50 µL water and 870 µL Methyl tert-butyl ether (MTBE) and vortexed for 1 h to extract the metabolites. Water (250 µL) was added to induce the separation of organic and aqueous phase and centrifuged at 15,000× *g* for 15 min at 4 °C. Lower aqueous and upper organic and layer (100 µL each) were vacuum dried in SpeedVac concentrator and stored till further analysis at −80 °C. Samples were reconstituted in 15% methanol (50 µL) and kept in ice for 30 min, vortexed for another 30 min, centrifuged at 15,000× *g* for 15 min at 4 °C and supernatant collected and were subjected to metabolomic analysis by using LC-MS platform.

#### 4.6.2. Metabolomics Measurement

The metabolomics data was acquired on the orbitrap fusion mass spectrometer (Thermo Scientific, Waltham, MA, USA) equipped with heated electrospray ionization (HESI) source. Data were acquired on positive and negative mode at 120,000 mass resolution in MS mode and 30,000 resolution in data dependent MS2 scan mode. Spray voltage of 4000 and 35,000 volt were used for positive and negative mode respectively. Sheath gas and auxiliary gas was set to 42 and 11 respectively. Mass scan range of 50–1000 *m*/*z*, automatic gain control (AGC) target at 200,000 ions and maximum injection time was 80 ms for MS and AGC target was 20,000 ions and maximum injection time 60 ms for MSMS was used. Extracted metabolites were separated on UPLC ultimate 3000 using HSS T3 column (100 × 2.1 mm i.d, 1.7 µm, waters) maintained at 40 °C temperature. The mobile phase A was water with 0.1% formic acid and mobile phase B was acetonitrile with 0.1% formic acid. The elution gradient used is as follows: 0 min, 1% B, 1 min, 15% B, 4 min, 35% B, 7 min, 95% B, 9 min, 95% B, 10 min, 1% B and 14 min, 1% B. The flow rate was 0.3 mL/min and sample injection volume was 5 µL. Pool quality control (QC) sample was prepared by collecting 10 µL from each sample and was run after every five samples to monitor retention time shift, signal variation and drift in mass error [95].

#### 4.6.3. Data Processing

All acquired data has been processed using Progenesis QI software (Waters Corporation, Milford, MA, USA) using default setting. The untargeted metabolomics workflow of Progenesis QI was used to perform retention time alignment, feature detection, elemental composition prediction and database search. Identification of metabolite was done on the basis of in house metabolite library with accurate mass, retention time and fragmentation pattern information match. Additionally, spectral data matching with mzcloud and mass bank for the fragmentation match for identification of metabolites were also used. Metabolomics data were normalized by sum and pareto scaled before multivariate analysis. Relative fold-change values in metabolite expression analysis were calculated for each treated samples with respect to the untreated time-matched control (WT) for further differential analysis. Fold change values were log transformed for clearer representation in the heat map analysis. Statistical analysis were performed by two-way ANOVA followed by a two-stage linear step-up procedure of Benjamini, Krieger, and Yekutieli to correct for multiple comparisons by controlling the False Discovery Rate (<0.05).

#### 4.6.4. Statistical Analysis

Data have been presented as Mean ± SD. Independent unpaired Student “*t*” test was used for comparison as appropriate using GraphPad Prism 8.0.2 software. More than two groups were compared by one-way analysis of variance (ANOVA) followed by post hoc Tukey’s multiple comparison test or Dunnett’s test. Differences were considered statistically significant at *p* < 0.05. For the correlation analysis, Pearson correlation coefficients were calculated and *p* value was corrected according to the Benjamini–Hochberg correction for multiple comparisons, with a false discovery rate *<* 0.05.

## Data Availability

All data used in this study are present in the main text and Supplementary Materials.

## Supplementary Materials

The following are available online at https://www.mdpi.com/article/10.3390/ijms23010195/s1.
