# Supplementary material for: Modulation of Insulin Resistance, Dyslipidemia and Serum Metabolome in iNOS Knockout Mice following Treatment with Nitrite, Metformin, Pioglitazone, and a Combination of Ampicillin and Neomycin
Source: Int J Mol Sci. 2021 Dec 24;23(1):195. doi: 10.3390/ijms23010195 (PMC8745663; doi:10.3390/ijms23010195)
Supplement: Supplementary file 1 [file ijms-23-00195-s001.zip › ijms-1465933-supplementary.pdf]

Supplementary figures

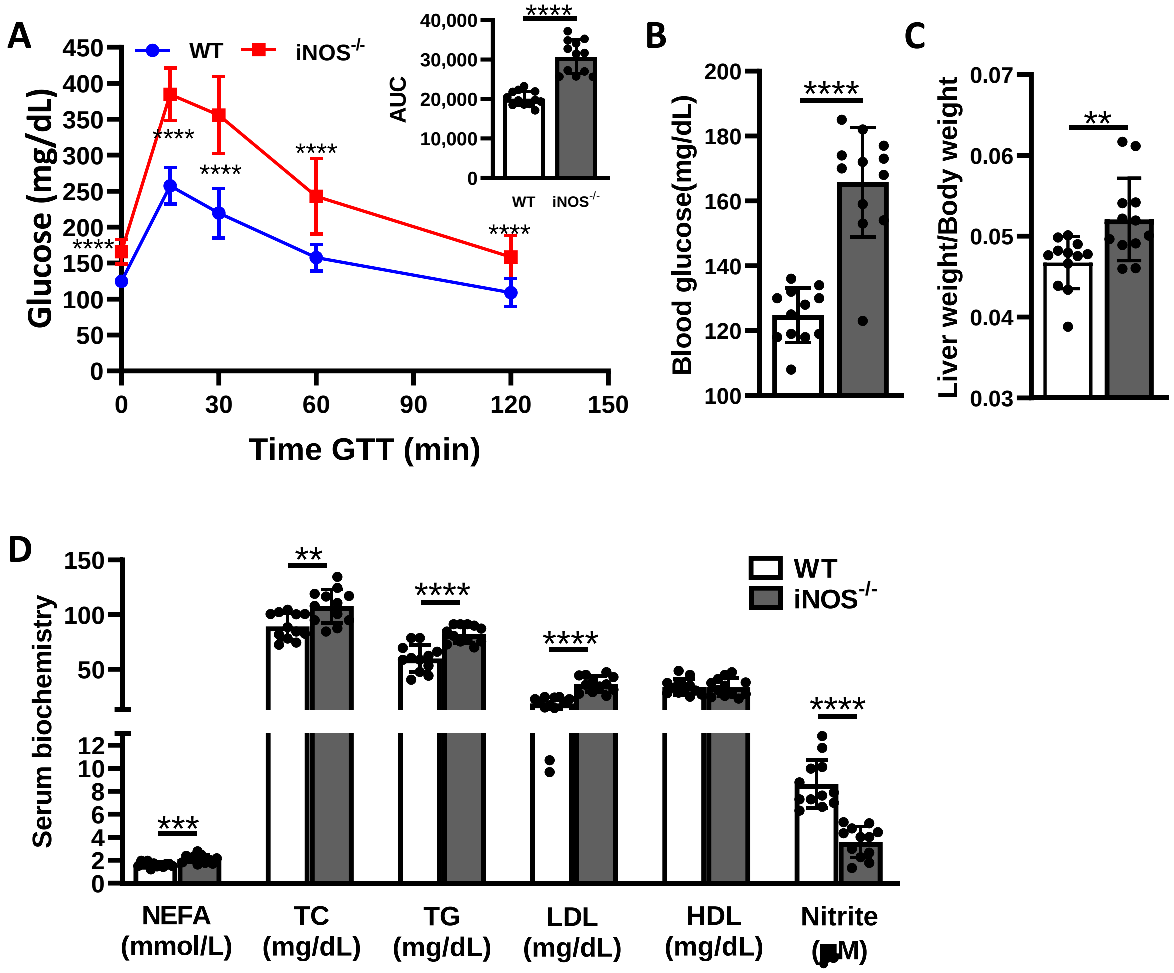

Figure S1: Systemic glucose intolerance and dyslipidemia in iNOS$^{-/-}$ mice. Systemic glucose and lipid homeostasis in iNOS$^{-/-}$ mice as compared to WT. (A) Intraperitoneal glucose tolerance test (GTT) with AUC calculated from IPGTT data, (B) Fasting blood glucose levels, (C) Relative liver weight ratio and (D) Serum lipids and total nitrite levels. Data are represented as mean ± SD (n=12). $^{**}p<0.01$, $^{***}p<0.001$ and $^{****}p<0.0001$ vs WT.

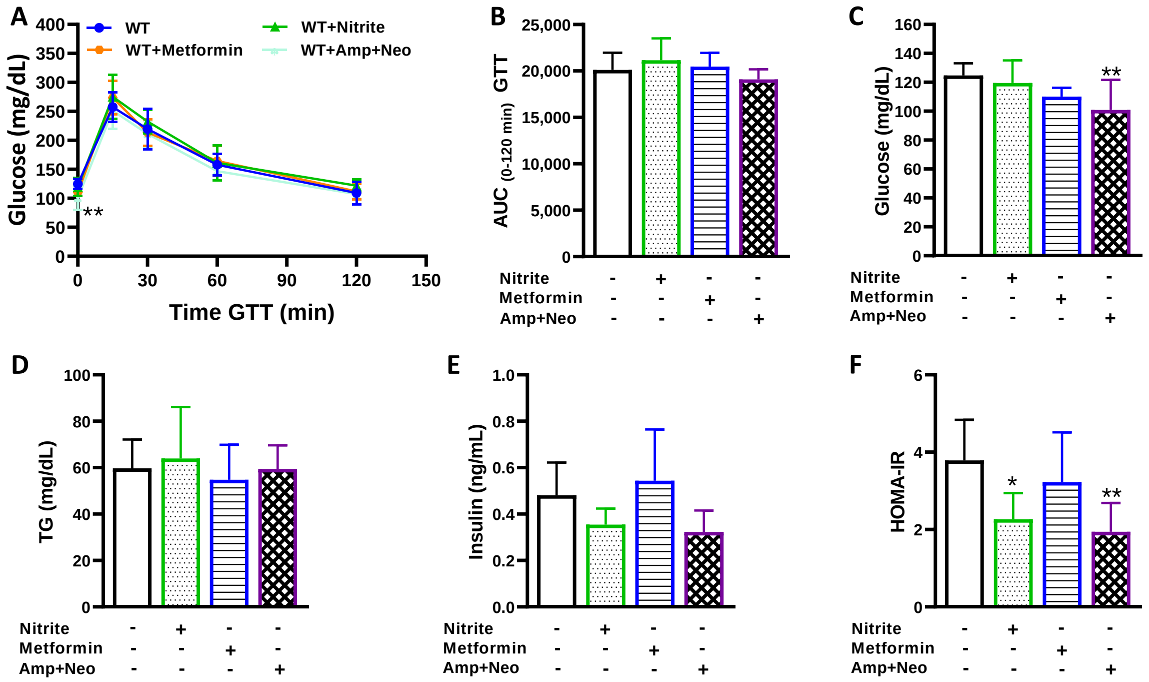

Figure S2: Effect of nitrite, metformin, and a combination of ampicillin and neomycin on systemic glucose, insulin and lipid homeostasis in WT mice. Systemic glucose, insulin and lipid homeostasis in WT mice upon treatment with nitrite, metformin and ampicillin/neomycin combination. (A) Intraperitoneal glucose tolerance test (GTT), (B) Area under the curve (AUC) calculated from IPGTT data, (C) Fasting blood glucose levels, (D) Serum triglycerides, (E) Fasting serum insulin levels and (F) Index of insulin resistance: HOMA-IR. Data are represented as mean ± SD (n=5-12). *p<0.05 and **p<0.01 vs WT.

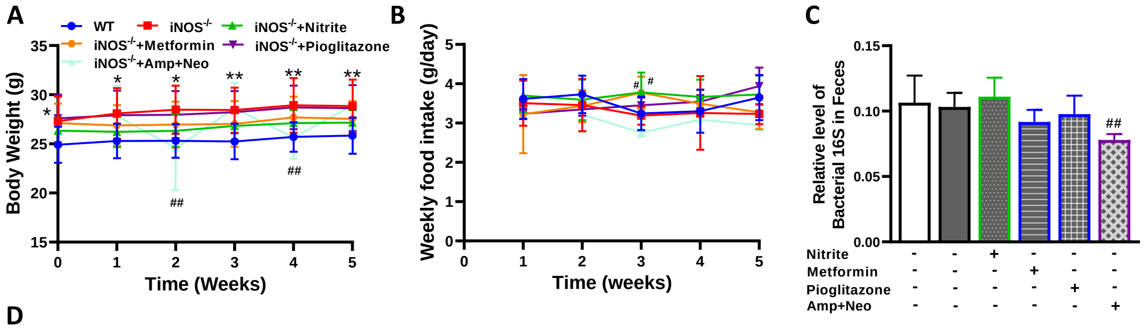

D

Parameters	WT	iNOS-/-	iNOS-/- + Nitrite	iNOS-/- + Metformin	iNOS-/- + Pioglitazone	iNOS-/- + Ampicillin + Neomycin
Heart weight ratio	0.005546 ± 0.0004453	0.005645 ± 0.0004009	0.005554 ± 0.0003166	0.005458 ± 0.0007373	0.005723 ± 0.0007102	0.004939 ± 0.0002616#
Spleen weight ratio	0.004168 ± 0.001959	0.003848 ± 0.001877	0.003818 ± 0.001363	0.004579 ± 0.001440	0.004368 ± 0.001404	0.003384 ± 0.0007613
Kidney weight ratio	0.0146 ± 0.001254	0.01392 ± 0.0008505	0.01314 ± 0.001878	0.01325 ± 0.0009299	0.01272 ± 0.001355	0.01332 ± 0.001965
BAT weight ratio	0.003509 ± 0.0006808	0.004080 ± 0.001755	0.004114 ± 0.001409	0.004770 ± 0.0008898	0.006414 ± 0.0009762##	0.003981 ± 0.0009295
Colon weight ratio	0.02033 ± 0.003194	0.01950 ± 0.005326	0.02040 ± 0.003045	0.02284 ± 0.004043	0.01612 ± 0.002670	0.02259 ± 0.002083
HOMA-B	56.38 ± 41.43	94.97± 51.20	43.50 ±22.92##	101.4± 38.41	66.60 ± 31.69	78.78 ± 38.28

Figure S3: Effect of nitrite, metformin, pioglitazone, and a combination of ampicillin and neomycin on gross parameters, food intake and bacterial 16S levels in iNOS$^{-/-}$ mice. (A) Body weight curve from the initiation to study termination, (B) Weekly food consumption from initiation to study termination (C) Relative level of bacterial 16S rRNA gene and (D) Relative tissue weights and HOMA-B index. Data are represented as mean ± SD (n≥5). *p<0.05 and **p<0.01 vs WT; $^{\#}$p<0.05 and $^{\#\#}$p<0.01 vs iNOS$^{-/-}$.

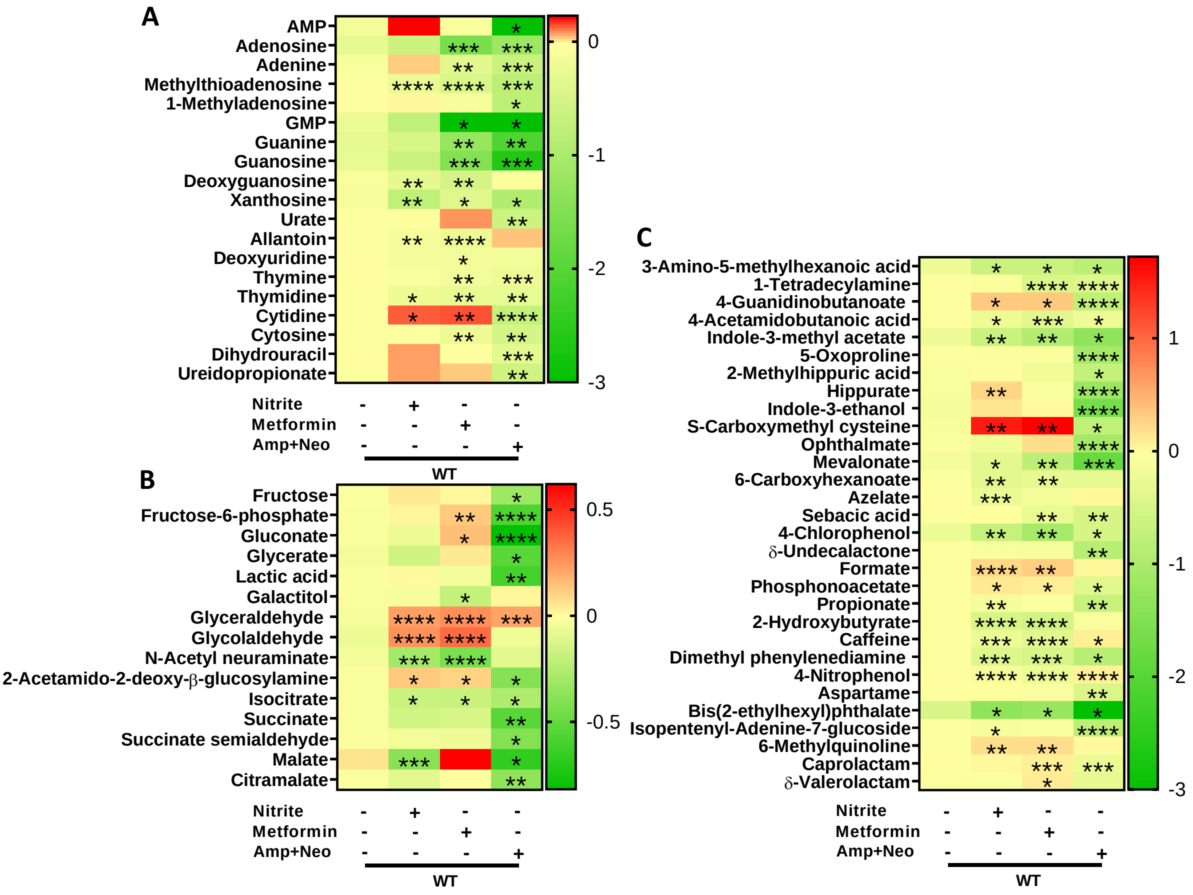

Figure S4: Alterations in the serum metabolome of WT mice following treatment with various interventions. Serum metabolomic analysis in chow fed WT mice with or without nitrite, metformin and ampicillin/neomycin combination treatment. Heat map of differential metabolites found by metabolomics analysis related to (A) Nucleic acids metabolism, (B) Carbohydrate metabolism and (C) Miscellaneous/ microbiota derived metabolites. Data are represented as mean (n≥5). *p<0.05, **p<0.01, ***p<0.001, ****p<0.0001 vs WT.

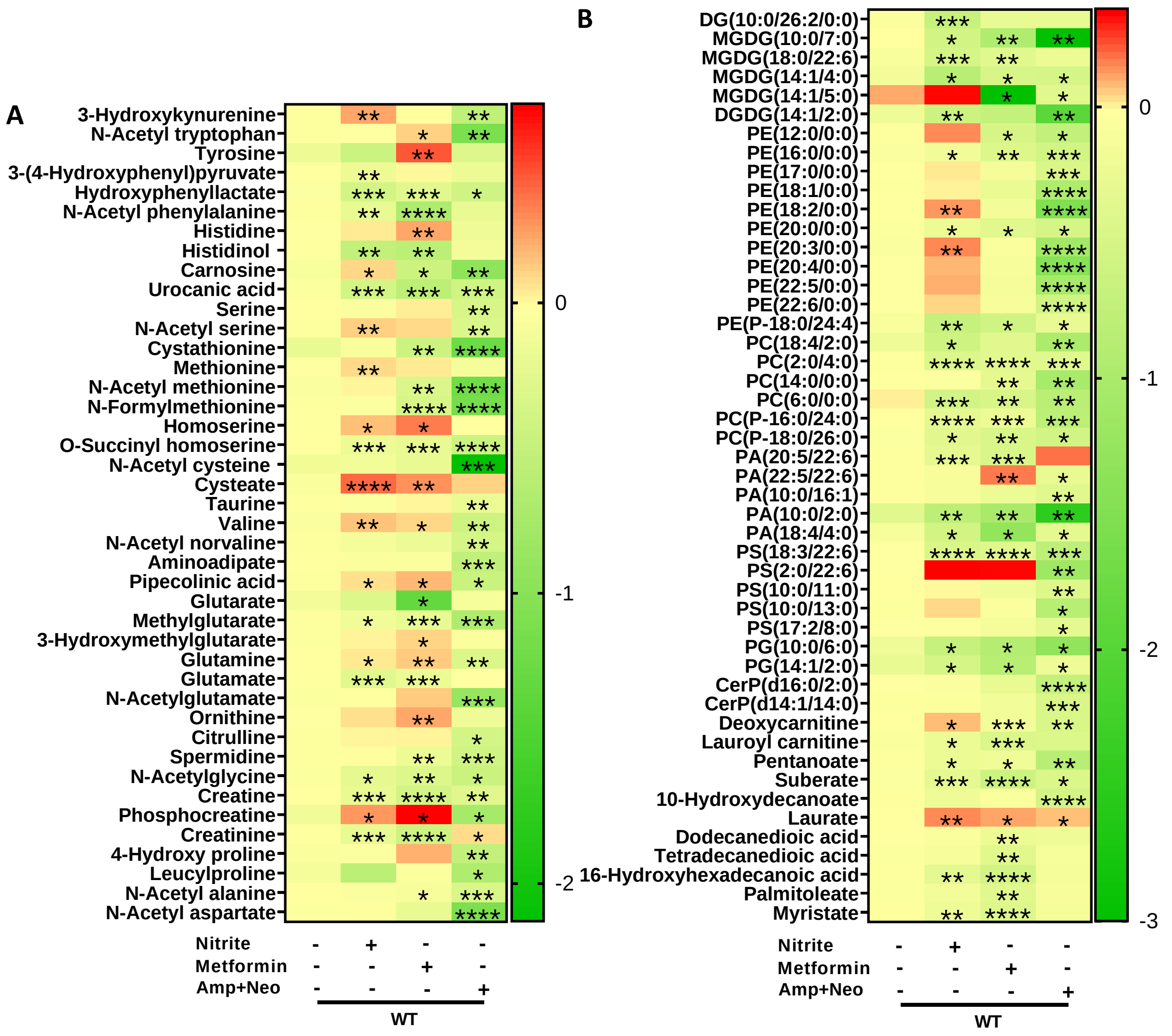

Figure S5: Alterations in the amino acid and lipid metabolites in WT mice following treatment with various interventions. Serum metabolomic analysis in chow fed WT mice with or without nitrite, metformin, and ampicillin/neomycin combination treatment. Heat map of differential metabolites found by metabolomics analysis related to (A) Amino acids metabolism and (B) Lipid metabolism. Data are represented as mean (n≥5). *p<0.05, **p<0.01, ***p<0.001, ****p<0.0001 vs WT.

Supplementary Tables

Parameters	WT	WT + Nitrite	WT + Metformin	WT + Ampicillin + Neomycin
NEFA (mmol/L)	1.611 ± 0.2176	1.751 ± 0.2234	1.654 ± 0.2421	1.618 ± 0.1598
TC (mg/dL)	89.17 ± 11.82	69.38 ± 25.42	62.57 ± 16.27*	85.1 ± 17.69
LDL (mg/dL)	18.65 ± 5.296	23.91 ± 3.342	23.79 ± 4.379	19.84 ± 4.085
HDL (mg/dL)	33.76 ± 7.222	36.29 ± 2.313	36.08 ±3.245	26.88 ± 10.64
Nitrite (μM)	8.622 ± 2.096	7.469 ± 1.705	6.082 ± 1.595	6.947 ± 1.633
HOMA-B	56.38 ± 41.43	53.9 ± 8.849	94.37 ± 44.17	60.21 ± 14.90
QUICKI	0.316 ± 0.01322	0.3397 ± 0.01426	0.327 ± 0.02766	0.3501 ± 0.02293**
Liver weight ratio	0.04674 ± 0.003232	0.05017 ± 0.008075	0.05195± 0.004364	0.04305 ± 0.005735
Heart weight ratio	0.005546 ± 0.0004453	0.005099 ± 0.0004319	0.005222 ± 0.0002555	0.00462 ± 0.00051***
Spleen weight ratio	0.004168 ± 0.001959	0.003208 ± 0.0008351	0.003233 ± 0.0003083	0.00361 ± 0.001279
Kidney weight ratio	0.0146 ± 0.001254	0.01345 ± 0.001395	0.01404 ± 0.0009087	0.01382 ± 0.002700
eWAT weight ratio	0.009354 ± 0.001715	0.007605 ± 0.002869	0.008167 ± 0.003132	0.01022 ± 0.001875
BAT weight ratio	0.003509 ± 0.0006808	0.002887 ± 0.0005232	0.004285 ± 0.002101	0.005426 ± 0.002666
Small intestine weight ratio	0.05748 ± 0.009052	0.09655 ± 0.02413***	0.06197 ± 0.002790	0.05962 ± 0.01017
Caecum weight ratio	0.02465 ± 0.003601	0.02388 ± 0.003960	0.02967 ± 0.003259	0.04336 ± 0.01033***
Colon weight ratio	0.02033 ± 0.003194	0.02008 ± 0.003845	0.02385 ± 0.0009760	0.01856 ± 0.001937

Table S1: Effect of nitrite, metformin, and a combination of ampicillin and neomycin on lipid and insulin homeostasis, and tissue weights in WT mice. Data are represented as mean ± SD (n≥5). $^{*}p<0.05$, $^{**}p<0.01$ and $^{***}p<0.001$ vs WT.
